# A streptavidin–biotin system combined with magnetic actuators for remote neuronal guidance

**DOI:** 10.1186/s13036-023-00359-3

**Published:** 2023-06-20

**Authors:** Dafna Rivka Levenberg, Eli Varon, Ganit Indech, Tal Ben Uliel, Lidor Geri, Amos Sharoni, Orit Shefi

**Affiliations:** 1 Faculty of Engineering, Bar-Ilan University, 5290002 Ramat Gan, Israel; 2 Bar-Ilan Institute of Nanotechnology and Advanced Materials, Bar-Ilan University, 5290002 Ramat Gan, Israel; 3 Gonda Multidisciplinary Brain Research Center, Bar-Ilan University, 5290002 Ramat Gan, Israel; 4 Department of Physics, Bar-Ilan University, 5290002 Ramat Gan, Israel

**Keywords:** Magnetic manipulation, Neuronal organization, Streptavidin–biotin, Magnetic particles

## Abstract

The ability to control neuronal mobility and organization is of great importance in developing neuronal interfaces and novel therapeutic approaches. An emerging promising method is the manipulation of neuronal cells from afar via magnetic forces. Nevertheless, using magnetic iron oxide nanoparticles as internal actuators may lead to biotoxicity, adverse influence on intracellular processes, and thus requires prerequisite considerations for therapeutic approaches. Magnetizing the cells via the incorporation of magnetic particles that can be applied extracellularly is advantageous. Herein, we have developed a magnetic system based on streptavidin–biotin interaction to decorate cellular membrane with magnetic elements. In this model, superparamagnetic microparticles, coated with streptavidin, were specifically bound to biotinylated PC12 cells. We demonstrated that cell movement can be directed remotely by the forces produced by pre-designed magnetic fields. First, using time lapse imaging, we analyzed the kinetics of cell migration towards the higher flux zone. Next, to form organized networks of cells we designed and fabricated micro-patterned magnetic devices. The fabricated devices were composed of a variety of ferromagnetic shapes, sputter-deposited onto glass substrates. Cells that were conjugated to the magnetic particles were plated atop the micro-patterned substrates, attracted to the magnetic actuators and became fixed onto the magnetic patterns. In all, our study presents a novel system based on a well-known molecular technology combined with nanotechnology that may well lead to the expansion of implantable magnetic actuators to organize and direct cellular growth.

**Supplementary Information:**

The online version contains supplementary material available at 10.1186/s13036-023-00359-3.

## Introduction

The ability to control and design neural network formation by targeting cells toward specific sites is of great importance in the field of biomedicine and tissue engineering [25]. The capability of directing cells has many potential implications in the field of biosensors and cell-based therapies. In recent years magnetic field-based manipulations of biological elements have been progressively developed due to clear advantages,magnetic fields can be activated remotely and be well controlled, are non-invasive, and can penetrate biological matter [5]. Previously, the use of magnetic thin films as bioselective logic for the separation of magnetized objects has been shown suggesting various profound applications [16]. Recently, magnetic manipulation of micro- and nano-objects has shown to be promising with numerous implications in potential therapeutics, specifically neural regeneration therapies [8]. The use of magnetic particles in neural network formation typically involves the labelling of cells internally [2, 13, 18–20]. Internal nanoparticles can undesirably affect biomolecular pathways at the cellular level including modulation of cell death, toxicity effect of metal core, and overall have very few in vivo studies established [17, 26, 31]. Furthermore, when using magnetic nanoparticles for cell magnetization applications, the magnetization is much lower than that could be achieved by magnetic microparticles (MMPs), resulting in weaker forces acting on the cells [30]. Hence, we aim to label the outer membrane with MMPs to circumvent these barriers.

Using magnetic particles for microfluidic cell manipulation, also known as “magnetophoresis”, is an appealing technology [32]. Since the magnetization of the magnetic particles is greater than its surrounding medium, cell-magnetic particle conjugates can be magnetized under external magnetic fields and consequently move towards the location of magnetic field maxima [7]. When the applied magnetic field is strong enough, the MMPs reach saturation. In the case of weak magnetic fields, the MMPs demonstrate a linear response to the applied magnetic field [1]. In both regimes, the total magnetic moment is proportional to the number of conjugated particles [3]. Here, we used both strong and weak magnetic fields to analyze cells’ velocity and magnetic response in our magnetic streptavidin biotin-based system for cell positioning.

In the present study, we propose an approach for physical neuronal guidance. We base our study on a previously developed technology that utilizes the strong non-covalent interaction of biotin-streptavidin with streptavidin-coated superparamagnetic microparticles [4, 10, 11]. Initially, PC12 cell membranes were coated with biotin followed by attaching streptavidin-coated superparamagnetic microparticles. This converted the cells into sensitive magnetic units that can be isolated by magnetic gradients. Following this step, we designed and fabricated a magnetic platform embedded with micropatterned ferromagnets and nanometric heights. The PC12 cells that were magnetically labeled were attracted and migrated towards the high magnetic flux zones and across the magnet. We specifically have chosen superparamagnetic particles to prevent aggregation of the cells following the magnetic actuation. Next, we measured the magnetophoresis based on the cell velocity due to magnetic particles attached to the membrane. Our study establishes an effective magnetic-based actuator to detect and direct neuronal cells. This platform can be tailored and designed based on the various magnetic patterns and coatings we fabricate. In all, we offer a method for non-invasive remote cell patterning and organization relevant for neural network formation.

## Results and discussion

**Fig. 1:**
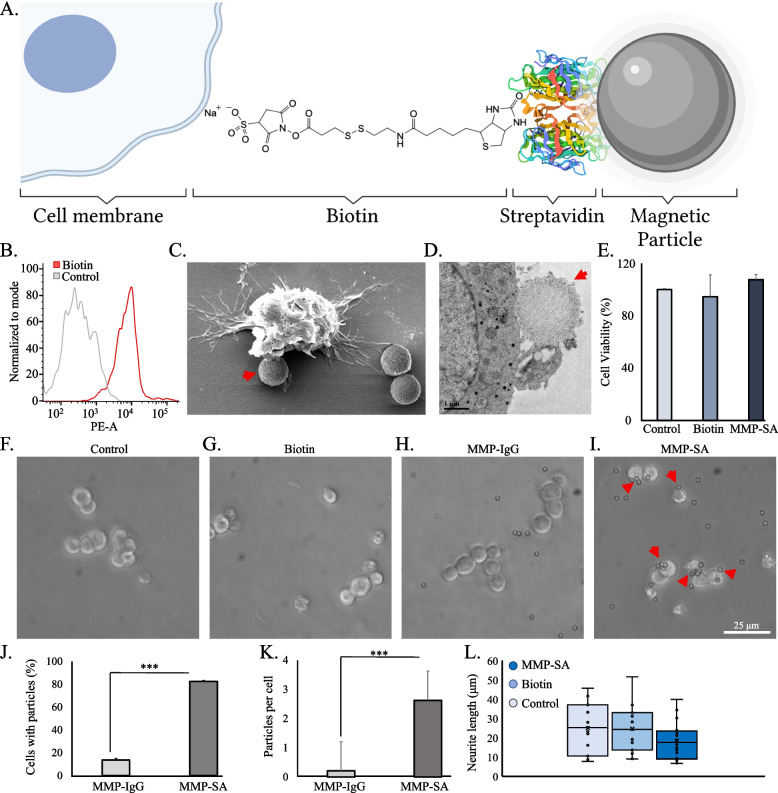
Interaction of biotin coated PC12 cells with streptavidin magnetic particles. **A** Illustration of biotinylated cells conjugated to streptavidin magnetic particles (not in scale). **B** Flow cytometry CY3 representative results. **C** HR-SEM images of biotinylated cells labeled with MMP-SA. **D** TEM images of biotinylated cell membrane labeled with MMP-SA. **E** XTT cell viability assay. The cell viability was calculated after 72 h. Results are normalized to control. **F**-**I** Phase contrast images of PC12 cells with different treatments. **J** Percent of cells (*n* = 102) conjugated to particles (%). **K** The average number of particles per cell (*n* = 102). **L** Average neurite length of PC12 cells. The average quantification of three experiments is presented in the plots (mean ± STDEV). There were significant differences in some of the experiments. ***P < 0.001

We used commercial superparamagnetic microparticles (MMP) which are 2.8 µm in diameter and coated with a monolayer of recombinant streptavidin covalently coupled to the surface, as mediators to exert magnetic gradient forces on the biotinylated cells (Fig. 1A). Thereafter, the cells were placed atop the magnetic actuators for neuronal guidance and network formation. The configuration of the cells conjugated to streptavidin magnetic particles are described herein.

### Labelling PC12 cells with biotin and streptavidin magnetic particles

The purchased colloidal magnetic microparticles were covalently coupled with monolayer of streptavidin (MMP-SA) or polyclonal sheep anti-rabbit IgG (MMP-IgG) for control tests (see experimental section). We sought to confirm via high resolution scanning electron microscope (SEM) and transmission electron microscopy (TEM) the actual binding and interaction of biotinylated cells to streptavidin and the magnetic complex configuration. Streptavidin is a homo-tetramer with each subunit having a high affinity for biotin with equal affinity and a dissociation constant (K_d_) on the order of ≈10^−14^ mol/L that cannot cause cross-linking [9, 12]. Our hypothesis was that by using this strong binding externally on the cell membrane we can migrate cells towards an external magnetic field effortlessly. As a control, we used the same magnetic particle but coated with IgG, that doesn’t have affinity towards biotin. By adding uranyl acetate (UA) for sample preparation, the highly dynamic structure was fixed. TEM examinations (Figure S1) corroborated the morphology of the conjugated proteins and particles. Following, we coated PC12 cells with biotin after optimizing its concentration (Fig S2) and conjugated MMP-SA externally to the cell membrane. To validate that the PC12 cells were properly coated with biotin we added Cy3 streptavidin antibody, displaying a fluorescence signal and confirmed that biotin is attached to the cellular membrane (Fig. 1B, S2). As seen from the HR-SEM images, the MMP-SA and MMP-IgG were spherical with a rough surface form in both dispersions (Figure S3). Yet only the MMP-SA were found to be attached to the cell membrane (Fig. 1C, S3) We confirmed that the SEM images indeed contained the MMP-SA complex using a High-Resolution Scanning Electron Microscope-linked energy-dispersive X-ray (XHR-SEM–EDX) microanalysis unit. The EDX spectrum showed strong peaks of iron (Fe) validating that these circular structures are the MMP-SA (Figure S4). Also, we located the absorption of the MMP-SA position on the cell membrane using TEM which demonstrated no consequences or disruption to the membrane (Fig. 1D).

Labeling cells in vitro with magnetic particles offers the opportunity of controlling cell migration and interaction [11, 19, 22]. Effective external cellular attachment of the particles is one of the priority characteristics for this engineered MMP-SA complex. To transform the cells into magnetic sensitive units we used a two-step method which first labels the cell membrane with biotin and then binds streptavidin superparamagnetic particles onto the biotinylated membrane proteins. To observe whether the MMP-SA are non-toxic we incubated PC12 cells with 50 μg/ml MMP-SA for 24 h. We found that cells containing the MMP-SA complex grew similarly to the control cells and demonstrated no difference in cellular morphology, with no cytotoxic effect (Fig. 1E-I).

We measured the percentage of cells that were bound to magnetic particles. 12.7 ± 1.3 percent of biotinylated cells were found to be conjugated to MMP-IgG verifying the low affinity of biotin to IgG, in comparison to 82.3 ± 0.7 percent of biotinylated cells that were conjugated to MMP-SA (Fig. 1J). Following, we quantified the number of magnetic particles per cell. On average 0.2 MMP-IgG particles and 2.8 MMP-SA particles were bound to each cell. (Fig. 1K). This validated the multiple active targeting of binding interactions between streptavidin and biotin which doesn’t occur with IgG. Following, we measured the neurite length to confirm that this conjugation doesn’t disturb neuronal growth. We found that MMP-SA treated cells developed similar neurite lengths in comparison to control cells after 72 h (Fig. 1L, S5).

### PC12 cells responsiveness to the magnetic forces

**Fig. 2:**
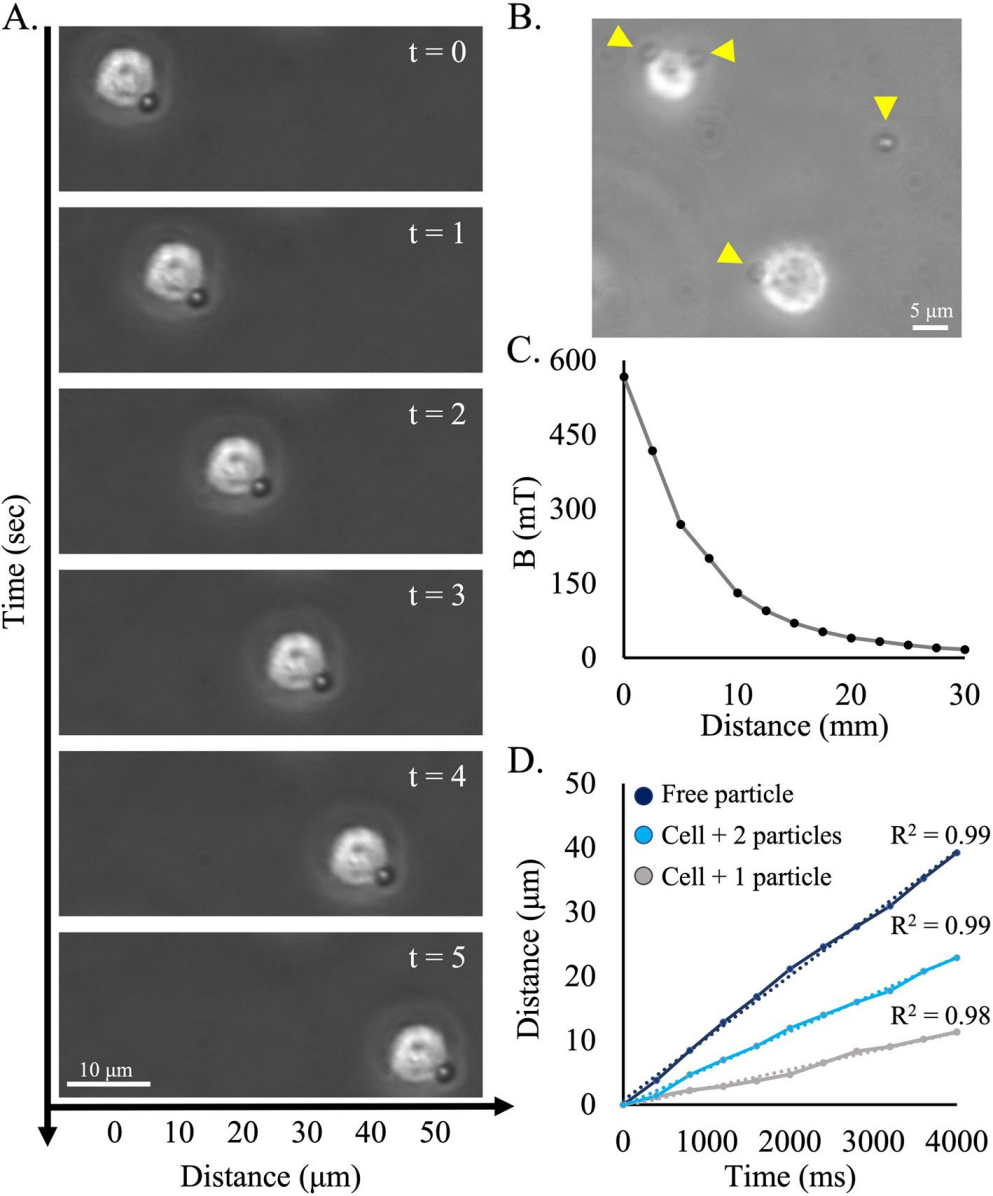
Live-cell time-lapse imaging and single-cell tracking of magnetically labeled PC12 cells. **A** Time-lapse images of a biotinylated cell conjugated to MMP-SA migrating to a permanent magnet. **B** Phase contrast projection images of PC12 cells in three different time points. **C** The magnetic field is stimulated by the permanent magnet on the side of the plate, according to measurements. **D** Linear function representing the distance (μm) over time (ms)

Following cell surface biotinylation and cell conjugation of MMP-SA, cells were suspended in a culture plate. A magnetic field was applied via an external permanent magnet that was positioned on one side of the plate. Automated time lapse imaging was used to directly observe the dynamic kinetic movement of the cells towards the magnet. Figure 2A displays the position of a cell that is labeled with a single MMP-SA with 1 s increments, showing that it moves within 5 s approximately 50 µm. In contrary, cells that were treated with MMP-IgG and placed in the external magnetic field stimulation displayed no movement. The speed of the cell was constant, indicating that the magnetic force, ${\overrightarrow{F}}_{mag}$, equaled the viscous drag force ${\overrightarrow{F}}_{drag}$.

We determined the alteration in speed among cells that were attached to different number of magnetic particles. This comparison can only be made within a single field of view where the magnetic field gradient is equally dispersed between the particles. Hence, we compared the speed of a single magnetic particle alone, to one magnetic particle conjugated to a cell, to two magnetic particles conjugated to a cell (Fig. 2B). Since all three cases showed a constant speed and are different only in their actual diameter (a cell is ~ 10 µm; a MMP is ~ 2.8 µm) or in their total magnetic moment (due to the number of conjugated particles), we assumed that the speed would change accordingly to maintain the balance between the magnetic and viscous drag forces. Hence, we expected to find that speed would be doubled between the cell with one particle to the cell with two particles and will be 3.5 times between the cell with one particle to the free particle (as the diameter/magnetic moment ratio). The actual measurements were in accordance with our hypothesis. (Fig. 2D) (for detailed calculation see supplementary material).

### Mechanical and physical properties of the magnetic substrate

Magnetic micro-patterns were fabricated by photolithography and sputter deposition of permalloy (Ni_0.8_Fe_0.2_) or CoFe/Pd multilayers, as described in the experimental section (based on [2, 13]). To magnetize the magnetic substrates in a controlled direction, an external magnet was placed close to the permalloy bars with the magnetic field aligned in the direction of the bars’ long axis, and for the CoFe/Pd magnetic patterns with the magnetic field oriented out of plane. For the patterned micromagnet substrates the induced magnetic field is much smaller than that of the external magnet, below the threshold for saturation [6]. Thus, one could assume a linear response for the MMPs, ${\overrightarrow{m}}_{particle}\propto {\overrightarrow{B}}_{micromagnet}$, which results in the magnetic force experienced by them [1]:

1 $${\overrightarrow{F}}_{mag}\propto \left({\overrightarrow{B}}_{micromagnet}\nabla \right){\overrightarrow{B}}_{micromagnet}$$

**Fig. 3:**
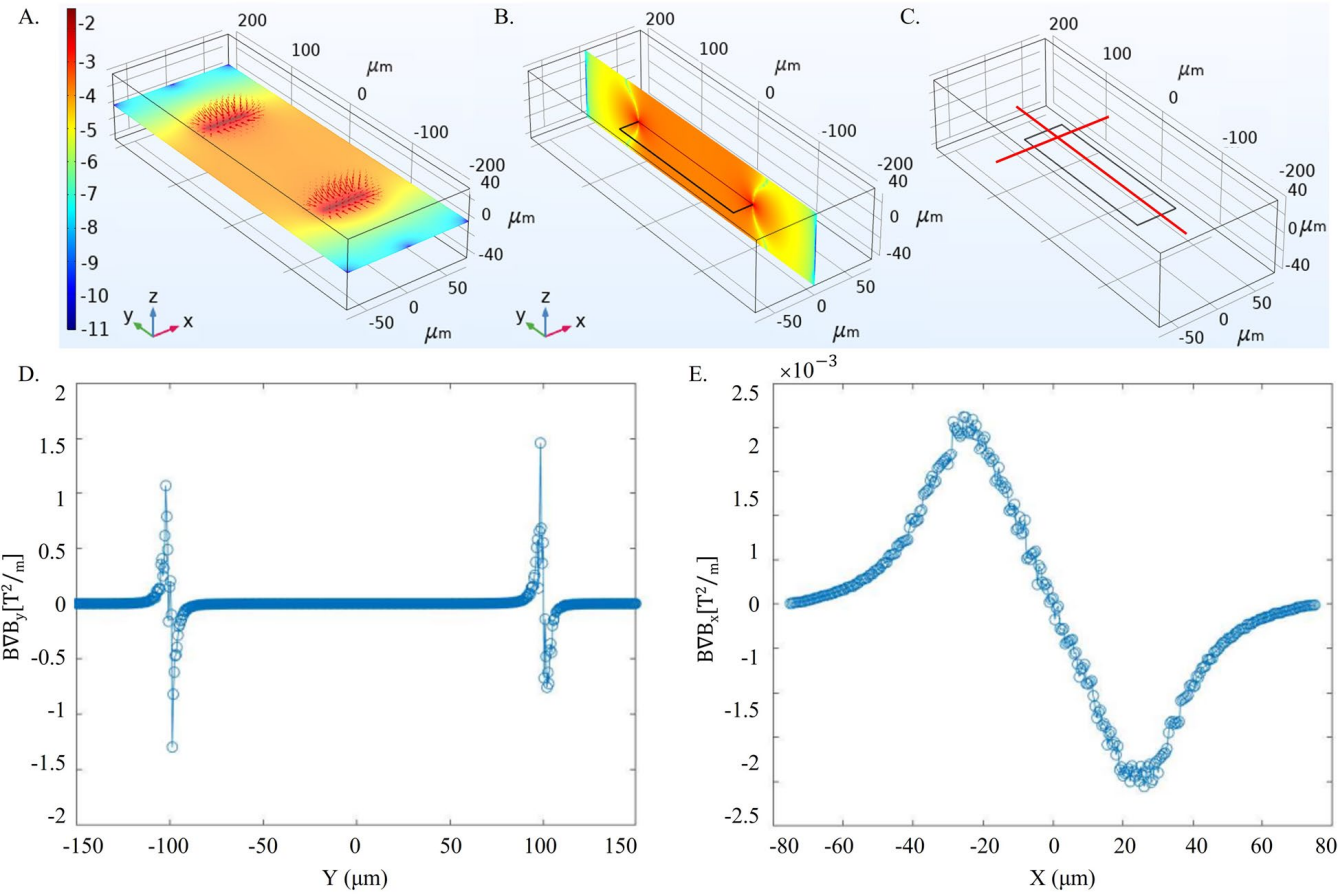
Simulations of magnetic micropatterns. **A** The magnetic flux density (B_norm_) in a log scale as simulated in COMSOL at 2 μm above a permalloy bar magnet with arrows indicating the magnetic force direction, showing strong magnetic attraction to the edges. **B** The magnetic flux density (B_y_) in a log scale as simulated in COMSOL for the YZ plane at x = 0 µm. **C**-**E** The magnetic attraction force magnitude at 2 µm above the magnet (**D**) along the Y axis at x = 0 µm and (**E**) along the X axis at Y = 80 µm (20 µm from the edges)

**Fig. 4:**
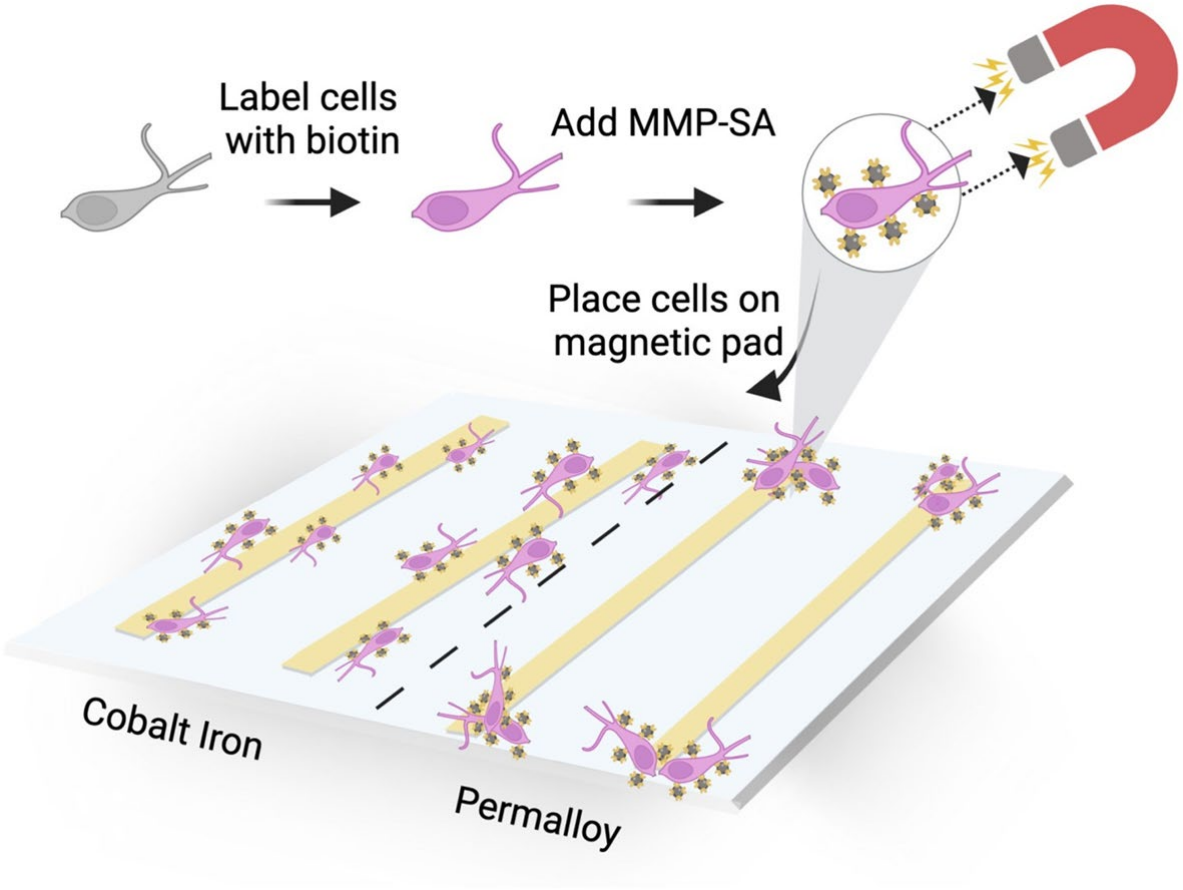
An illustration of neuronal migration to cobalt iron or permalloy magnetic actuators with different anisotropies

**Fig. 5:**
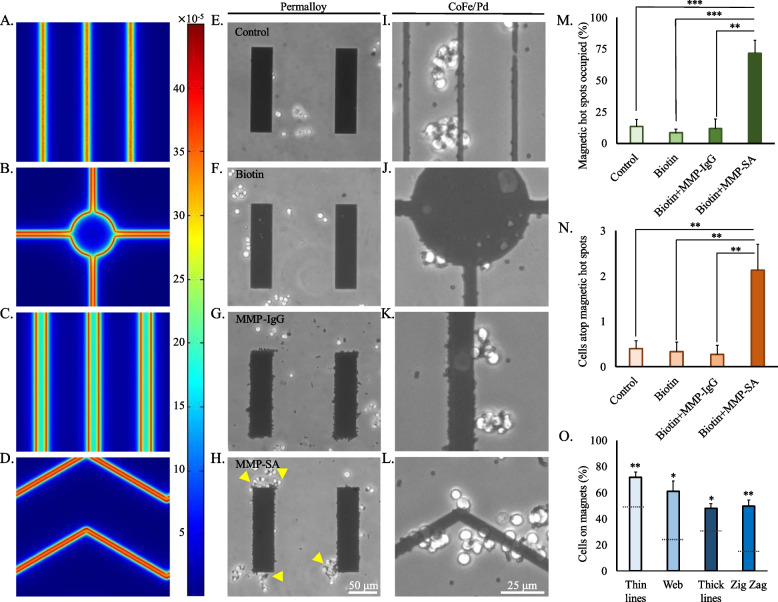
The efficiency of neuronal migration to cobalt iron and permalloy magnetic actuators. **A**-**D** Magnetic simulations of the induced magnetic flux density (B_norm_) at 2 μm above the magnet. Scale units are in Tesla. **E**-**L** Phase images of magnetized cells atop permalloy or CoFe/Pd magnetic patterns. **M** The number of occupied magnet bars and (**N)** the number of cells that reached the magnetic pattern. **O** Percentage of cells that reached the magnetic patterns. Dashed line represents the percentage of cells should reach the magnetic pattern randomly, regardless of the magnetic effect. The average quantification is presented in the plots (mean ± STDEV). There were significant differences in some of the experiments. *P < 0.05, **P < 0.01, ***P < 0.001

To examine the magnetic attraction of the permalloy bar we simulated and plotted the magnetic forces at 2 µm above the magnet, across lines in y and x direction (as shown in Fig. 3C) according to Eq. (1). Results are presented in Fig. 3D,E. It is noticeable that the force in the direction of the bar's long axis is much stronger than that of the short axis (in 3 orders of magnitude). Figure 3A presents the simulated norm of the magnetic flux density induced by a permalloy bar, which has in plane magnetization, in a logarithmic scale with arrows pointing the force direction, demonstrating two ‘hot spots’ that will attract magnetic particles. The magnetic flux density in $\widehat{y}$ direction for the YZ plane at $x=0 \mu m$ is presented in a logarithmic scale in Fig. 4B. We simulated the magnetic flux density of the various patterns, demonstrating magnetic fields in the scale of $<1 mT$ (Fig. 5A-D), which correspond with being in the linear region of the MMPs magnetic response.

### Magnetic actuators improve distant neuronal guidance

Next, we evaluated whether we could remotely manipulate the cellular migration using our magnetic actuators (Fig. 4).

To study the influence of the magnetic actuators on cells, cells were first placed atop the permalloy magnetic actuators. Following, we assessed the distribution of the cells along the magnetic patterns. Cells that were absent of magnetic particles or treated with MMP-IgG were uniformly dispersed throughout the culture dish (Fig. 5E-G). When cells were treated with MMP-SA, we attested to 71.6 ± 10.4 percent of magnetic hot spots occupied (Fig. 5H,M) with 2.1 ± 0.5 cells per magnetic pattern (Fig. 5N). Our results demonstrate that the magnetic patterns can exert substantial magnetic forces, acting on the cell membranes bound to particles and consequently prompting migration in the direction of the micro-magnets, specifically the magnetic ‘hot spots’ at the bar poles, as expected by in-plane magnetization.

Next, we compared the effect of the magnetic actuators made of CoFe/Pd multilayers which have a perpendicular magnetization easy axis; thus, are expected to attract magnetically along the entire pattern and not only at the poles. To quantify the localization effects, the number of cells landing atop the magnetic patterns was measured (Fig. 5O). In this analysis, all cells touching the pattern were counted. The percentage of cells that touched the magnets was compared to the percentage of cells we would expect to get from a uniform distribution, considering the ratio between the magnetic area and the total substrate area. Since the cells diameter is ≈10 µm the effective percentage of the magnetic area is larger than the nominal. Adding 10 µm to the edges of the magnetic shapes leads to an effective magnetic area of 51%, 25%, 33%, 18% for the thin lines, web, thick lines, and zig zag, respectively. Therefore, at uniform distribution, for example, only 5% of cells are expected to land on (or touch) the thin lines. The results of one-sample proportion test are displayed in Fig. 5O.

## Conclusion

In the present study, we established the application of well-regulated magnetic fields on biotinylated PC12 cells conjugated to MMP-SA to direct cell migration. Employing an external magnetic field caused PC12 cells to travel toward the magnet. We showed that this migration is linear and proportional to the number of conjugated particles since the velocity is constant and affected by the total magnetic moment. Additionally, we designed and fabricated micropatterned devices with permalloy or cobalt iron/palladium actuators that stimulate localized magnetic fields that may be controlled due to the actuator’s geometry and material.

Many research groups have formerly developed methods to give living cells magnetic properties to manipulate cell mobility [11, 14, 16, 19, 20, 22, 23, ]. Adapting magnetic manipulations as a method to control cell motility requires multiple bio-physical considerations [15, 24, 31]. To drastically increase the magnetic response of the cells we introduce magnetic microparticles that are tightly bound to the external cellular membrane maximizing the magnetic effect (e.g., the volume of a single 2.8 µm MMP is equal to ~ 150,000 magnetic nanoparticles with 50 nm diameter).

Organizing cells in a controlled manner is of great importance for tissue engineering and future therapies. Moreover, engineering neuronal networks is a fundamental step towards artificial brain organoids on chips, bio-hybrid implantations, or engineered neuronal circuits for bio-electronic computational devices [16, 27–29]. The development of different fabricated magnetic shapes can be used for various applications leading to controllable networks. To note, the method of conjugating the cells with MMPs should also be adjusted to the biological system and need. For example, different applications may require different levels of magnetization and control over the number of MMPs per cell. Moreover, a need for reversibility may require the ability to cleave the bond between streptavidin and biotin which is still a challenge in living cells. Nevertheless, the advantages of our combined technologies surpass the disadvantages, offering many applications derived from this technique to be explored in the future.

Overall, we suggest the application of this micropatterned magnetic device combined with magnetically labeled cells as a system for directing populations of neurons in a microscopic resolution-controlled manner. This may pave the way for the design of advanced neurochip interfaces and the future development of therapeutic applications.

## Experimental section

### TEM

For the particle measurements, carbon type A grid was glow discharged with a EmiTech K100 machine then 3 μl of MMP-IgG or MMP-SA was loaded on the grid. After 1 min the sample was blotted and access material then stained with Uranyl acetate 1% for 30 s and rinsed with double distilled water then air dried. For cellular and particle TEM samples, PC12 cells were seeded and incubated on 10 mL culture plate at a density of 3 × 10^6^ per plate for 24 h before the experiment. The cells were then treated with biotin, MMP-IgG, MMP-SA in serum-free media. The medium containing the biotin, MMP-IgG, MMP-SA was then discarded, and the cells were completely washed with PBS three times. The cells were then fixed for 2 h in Karnovsky-fixative (2.5% glutaraldehyde with 2.5% paraformaldehyde) in a 0.1 M sodium cacodylate buffer (pH 7.4) and washed with 0.1 M sodium cacodylate buffer. The cells were postfixed in 1% OsO_4_, 0.5% K 2 Cr_2_ O_7_, 0.5% K 4[Fe(CN)_6_]in 0.1 M cacodylate-buffer (pH 7.4) for 1 h at room temperature, then washed twice with 0.1 M cacodylate-buffer, followed by rising with DDW three times. Cells were then stained with 2% uranyl-acetate for 1 h, washed with DDW and dehydrated in Ethanol and embedded in Epon EMBED 812 (EMS). The resin was polymerized at 60 °C for 24 h. Ultrathin Sects. (70–90 nm) were obtained with a Leica ultracat (UC7) Ultramicrotome. Both methods samples where then inspected with a Tecnai G2 microscope (FEI –Teramo fisher) with an acceleration voltage of 120 kV. Images were taken using Digital Micrograph with a Mulitiscan Camera model 794 (Gatan) in different resolutions.

### SEM

For preparation of cellular and particle SEM samples, PC12 cells were seeded and incubated on 10 mL culture plate at a density of 3 × 10^6^ per plate for 24 h before the experiment. The cells were then treated with either biotin, MMP-IgG, MMP-SA, in serum-free media. The medium containing the biotin, MMP-IgG, or MMP-SA was then discarded and the cells were completely washed with PBS three times. The cells were then fixed for 2 h in Karnovsky-fixative (2.5% glutaraldehyde with 2.5% paraformaldehyde) in a 0.1 M sodium cacodylate buffer (pH 7.4) and washed with 0.1 M sodium cacodylate buffer, rinsed with double distilled water and dehydrated in Ethanol and then incubated in Hexamethyldisilazane for 10 min then air dried. Samples were then coated with gold with a Quarom coater inspected with a Magellan 400L (FEI–Teramo fisher) which is equipped with an oxford EDX detector. Images were taken at different resolutions.

### Imaging

Stable images and time-lapse live images were captured by a digital camera (Point Gray Chameleon3) coupled to a light microscope (Accuscope 3032 inverted microscope). Images were taken using phase contrast mode (magnification × 40, NA = 0.6).

### Kinetic calculations

The force a magnet attracts cells with MMPs depends on their relative position and the total magnetic moment of the microparticles on the cell. When applying an external magnetic field, the MMP experiences a magnetic force:

2 $${\overrightarrow{F}}_{mag}=\left({\overrightarrow{m}}_{\mathrm{particle}}\nabla \right)\overrightarrow{B}$$

where ${\overrightarrow{m}}_{\mathrm{particle}}$ is the magnetic moment of the particle and $\overrightarrow{B}$ is the magnetic field flux density. In the case of microparticles suspended in an aqueous solution, the total moment of the particle can be written ${{\overrightarrow{m}}_{particle}=V}_{m}M$, where ${V}_{m}$ is the volume of a particle and $M$ is the volumetric magnetization. When a MMP moves toward a magnet in a constant velocity, the magnetic force is balanced by the viscous drag force:

3 $${\overrightarrow{F}}_{drag}=3\pi \mu D{u}_{0}$$

where $\mu$ is the medium viscosity, $D$ is the particle or the cell diameter and ${u}_{0}$ is the particle or the cell velocity (Depends on whether you calculate the force acting on a particle or on a cell decorated with particles).

As the velocity of the cells and the particle is constant, when the volume of the magnetic particle is doubled in Eq. (2), the velocity in Eq. (3) should also be doubled to maintain the balance between the magnetic and the viscous drag forces. In the case of the free particle, the velocity should be multiplied by the same factor that the diameter decreased by.

### Magnetic simulations and calculations

We used the COMSOL Multiphysics 5.5 software to perform the modeling and magnetic -fields of different magnetic shapes and magnetic anisotropy directions with-no-currents physics to simulate the magnetic flux density. To overcome the wide scale of dimensions (height in nanoscale and width in microscale) we changed the height to micron and divided the magnetization in the same ratio. See Supporting Information for details of how the relative attraction force on the cells coated with superparamagnetic microparticles was estimated.

### Fabrication of ferromagnetic patterned substrates

The ferromagnetic patterns consisted of rectangles made of permalloy (Ni_80_Fe_20_) with a size of 200 × 50 μm^2^, a lateral spacing of 150 μm and a vertical spacing of 200 μm. The ferromagnetic patterns were prepared on precut glass slides 1 × 1 cm^2^. The glass slides were ultrasonically cleaned using acetone (5 min) and isopropanol (5 min), then dried by ultra-high purity (UHP) nitrogen. Patterns were fabricated by standard photolithography. AZ 1512H photoresist (MicroChemicals GmbH) was spin-coated on the glass substrate at 4500 rounds per min for 45 s, and baked at 100 °C for 60 s. Next, using maskless photolithography (MLA150, Heidelberg Inst.) the predesigned masks were transferred to the resist. The substrates were then developed for 60 s in B-351, diluted 1:4 in water, washed in distilled water for 45 s to stop the developer, and dried with UHP nitrogen gas. The samples were transferred to the deposition chamber and were cleaned with ion sputtering for 5 s prior to deposition. We deposit a 3 nm Cr adhesion layer followed by a 74 nm permalloy layer and a 3 nm Ti layer for the permalloy substrates (total height of ~ 80 nm). and 14 bilayers of Co_80_Fe_20_/Pd deposited with a ratio of 0.2 nm/1.0 nm respectively, followed by 2.0 nm of Pd capping layer for the CoFe/Pd substrates (total height of ~ 18 nm). Finally, the non-patterned photoresist was removed by soaking the sample in acetone for an hour and rinsing with isopropanol.

### Magnetic field measurements

A digital gaussmeter (Scientific Equipment Roorkee, DGM-204) was used to measure the magnetic field induced by the external permanent magnet.

### Cell culture

PC12 cells were grown in suspension in the RPMI medium supplemented with 10% horse serum (HS), 5% fetal bovine serum (FBS), 1% L-glutamine, 1% penicillin–streptomycin and 0.2% amphotericin, in a humidified incubator at 37 °C containing 5% CO_2_ (medium and supplements were purchased from Biological Industries, Israel). To induce differentiation, cells were seeded on plates coated with collagen type l and incubated for 24 h in serum reduced media (1% HS). Murine β-NGF (Peprotech, Israel) was then added to the medium (50 ng ml^−1^) as a free reagent. For the biotinylation, the cells were washed twice with ice-cold PBS and incubated for 40 min with 50 µg biotin per ml PBS at RT. For optimization of the biotinylation process we incubated the cells with 10, 50, 100 µg biotin per ml PBS at RT. The cells were then washed twice with ice-cold PBS and incubated with 50 μg/ml of M-280 streptavidin Dynabeads (Invitrogen) or M-280 sheep anti-rabbit IgG Dynabeads (Invitrogen) in serum reduced media at 37 °C for 30 min, to allow the particles to interact with the cells. For the magnetic patterning experiments, the cells were then plated in a 24 well plate containing a ferromagnetic patterned substrate.

### Flow cytometry studies

To confirm that cells were coated with biotin, Cy3-conjugated streptavidin (Jackson ImmunoResearch Laboratories, West Grove, PA, USA) was added and then analyzed using flow cytometry (BD LSRFortessaTM). For PC12 cell conjugation purposes, cells were treated with either biotin, MMP-IgG, MMP-SA, in serum-free media and were incubated in a 24-well culture plate at a density of 2 × 10^5^ per well. The cells were then collected. Samples were incubated in darkness for 5 min at room temperature and then analyzed using flow cytometry (BD LSRFortessaTM). The acquired data was analyzed using FlowJo software (Ashland, Oregon, USA).

### Cell viability assay

The viability of the cells was assessed using a colorimetric XTT assay. The assay is based on the ability of metabolically active cells to reduce tetrazolium salt XTT to orange-colored compounds of formazan. The intensity of the dye is proportional to the number of metabolically active cells. 2 × 10^4^ PC12 cells were seeded on 96-well plates, in the presence of biotin or biotin with MMP-SA or as PC12 cells alone. After 24 h of treatment exposure, XTT reaction solution (Biological Industries, Israel) was added to the medium and incubated for 5 h at 37 °C in triplicates. Absorbance was measured using a spectrophotometer (BioTek Synergy H1, Vermont USA) at 450 nm and 630 nm as background.

### Growth analysis

Analysis was performed on phase microscopy images of PC12 cells taken at day 3. Growth was assessed by measuring the mean neurites length. We used NeuronJ, an ImageJ plugin (US National Institutes of Health, Bethesda, MD, USA), which enables semi-automatic tracing of neurites and length measurements [21]. For each experiment, morphological parameters and statistics were measured for a total of 10 cells per treatment (PC12, PC12 + Biotin, PC12 + Biotin + MMP-SA).

### Statistical analysis

To examine the significancy between the control and the treated samples, we used an either a one-sample proportion test or two-sample t-test. The results are presented as mean values ± STDEV. A *p*-value of 0.05 was considered statistically significant.

### Supplementary Information

**Additional file 1: Figure S1.** Transmission electron microscopy of MMP-SA MMP-IgG. MMP-SA and MMP-IgG were stained with uranyl acetate solution, which enhances the electron density of hydrophilic areas.** Figure S2.** Flow cytometry CY3 measurement for the efficiency of different biotin concentrations for the biotinylation process of PC12 cells. The biotin concentration effect on the cell coating effectivity reaches saturation at 50 µg per ml.** Figure S3.** SEM images of PC12 after incubation alone or with Biotin, MMP-IgG, or MMP-SA. Bar = 10 μm.** Figure S4.** EDX analysis of PC12 cells treated with MMP-SA. Energy disperse spectroscopic image taken from PC12 cells treated with MMP-SAEnergy disperse spectroscopic spectra, the element weight percent of 89 C and Fe is displayed.** Figure S5.** Phase contrast images of PC12 cells with different treatments after 72 h. Bar = 10 μm.

## Data Availability

All data generated or analyzed during this study are included in this manuscript.
