# Supplementary material for: A streptavidin–biotin system combined with magnetic actuators for remote neuronal guidance
Source: J Biol Eng. 2023 Jun 20;17:40. doi: 10.1186/s13036-023-00359-3 (PMC10283188; doi:10.1186/s13036-023-00359-3)
Supplement: Supplementary file 1 — Additional file 1: Figure S1. Transmission electron microscopy of MMP-SA MMP-IgG. MMP-SA and MMP-IgG were stained with uranyl acetate solution, which enhances the electron density of hydrophilic areas. Figure S2. Flow cytometry CY3 measurement for the efficiency of different biotin concentrations for the biotinylation process of PC12 cells. The biotin concentration effect on the cell coating effectivity reaches saturation at 50 µg per ml. Figure S3. SEM images of PC12 after incubation alone or with Biotin, MMP-IgG, or MMP-SA. Bar = 10 μm. Figure S4. EDX analysis of PC12 cells treated with MMP-SA. Energy disperse spectroscopic image taken from PC12 cells treated with MMP-SAEnergy disperse spectroscopic spectra, the element weight percent of 89 C and Fe is displayed. Figure S5. Phase contrast images of PC12 cells with different treatments after 72 h. Bar = 10 μm. [file 13036_2023_359_MOESM1_ESM.docx]

A Streptavidin-Biotin System Combined with Magnetic Actuators for Remote Neuronal Guidance

Dafna Rivka Levenberg^1,2,3,†^, Eli Varon^1,2,3,†^ , Ganit Indech^2,4^, Tal Ben Uliel^1,2,3^, Lidor Geri^2,4^, Amos Sharoni^2,4,^ and Orit Shefi ^1,2,3,*^

^1^ Faculty of Engineering, Bar-Ilan University, Ramat Gan 5290002, Israel.

^2^ Bar-Ilan Institute of Nanotechnology and Advanced Materials, Bar-Ilan University, Ramat Gan 5290002, Israel.

^3^ Department of Physics, Bar-Ilan University, Ramat Gan 5290002, Israel.

^4^ Gonda Multidisciplinary Brain Research Center, Bar-Ilan University, Ramat Gan 5290002, Israel.

† Co-first author, these authors contributed equally to this work.

^*^ Correspondence: [orit.shefi@biu.ac.il](mailto:orit.shefi@biu.ac.il).

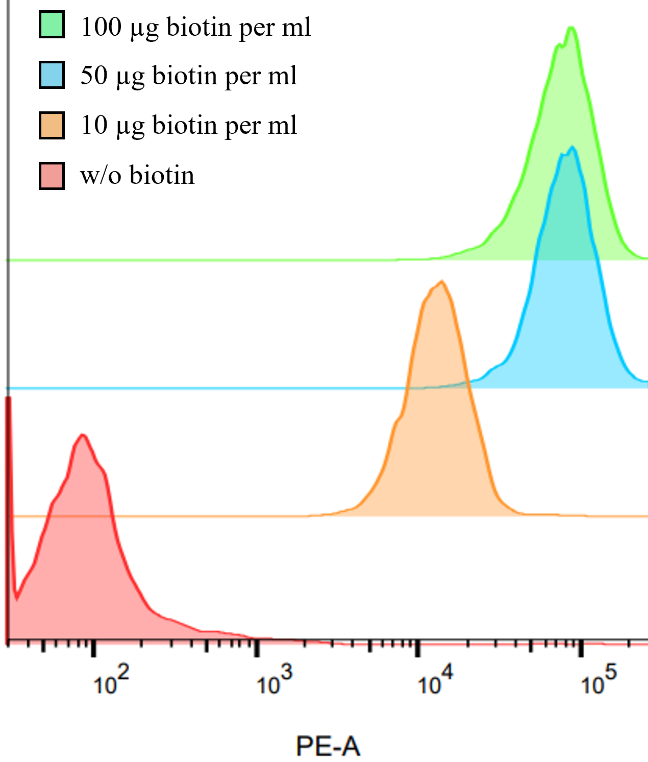
**Figure S1** | Transmission electron microscopy (TEM) of MMP-SA (A,C) MMP-IgG (B,D). MMP-SA and MMP-IgG were stained with uranyl acetate solution, which enhances the electron density of hydrophilic areas.

**Figure S2 |** Flow cytometry CY3 measurement for the efficiency of different biotin concentrations for the biotinylation process of PC12 cells. The biotin concentration effect on the cell coating effectivity reaches saturation at 50 µg per ml.

**Figure S3** | SEM images of PC12 after incubation alone or with Biotin, MMP-IgG, or MMP-SA. Bar = 10 μm.

**Figure S4** | EDX analysis of PC12 cells treated with MMP-SA. Energy disperse spectroscopic image taken from PC12 cells treated with MMP-SA (white box) (B) Energy disperse spectroscopic spectra, the element weight percent of 89 C and Fe is displayed.

**Figure S5** | Phase contrast images of PC12 cells with different treatments after 72 hr. Bar = 10 μm.

**Supplementary Material**

The magnetic force acting on a magnetic microparticle $\vec{F}_{mag}=\left( \vec{m}_{\mathrm{particle}}\nabla\right)\vec{B}$ is balanced by the viscous drag force $\vec{F}_{drag}=3\pi\mu Du_{0}$, i.e., $\vec{F}_{mag}=\vec{F}_{drag}$. This means that the speed would be doubled between the cell with one particle to the cell with two particles and will be 3.5 times between the cell with one particle to the free particle, detailed in the calculation below:

MMP volume^a^

Magnetization saturation

The magnetic gradient at x=2.5 cm (approximated distance between imaged cells and magnet)

For a free particle

$${\vec{\boldsymbol{F}}}_{\boldsymbol{x} \boldsymbol{magnetic}}\boldsymbol{=}\left( {\vec{\boldsymbol{m}}}_{\mathbf{particle}}\boldsymbol{\nabla} \right){\vec{\boldsymbol{B}}}_{\mathbf{x}}\boldsymbol{=}\left( \boldsymbol{V}_{\boldsymbol{m}}\boldsymbol{M\nabla} \right){\vec{\boldsymbol{B}}}_{\mathbf{x}}$$

$$V_{m}=\frac{4}{3}\pi r^{3}=\frac{4}{3}\cdot3.14\cdot\left( 1.4\cdot{10}^{-6} \right)^{3}\cong1\cdot{10}^{-17}\left[ m^{3} \right]$$

$$M=M_{saturation}=14kA/m=14\cdot{10}^{3}A/m$$

$$B_{x=2.5cm}-B_{x=2.25cm}=26-33\left[ mT \right]=-7[mT]$$

$$\nabla B_{x=2cm}=7[mT]/0.25[cm]=2.8[T/m]$$

$$\vec{F}_{x magnetic}=1\cdot{10}^{-17}\left[ m^{3} \right]\cdot14\cdot{10}^{3}\left[ A/m \right]\cdot2.8\left[ T/m \right]=3.9\cdot{10}^{-13}[N]$$

$${\vec{\boldsymbol{F}}}_{\boldsymbol{x} \boldsymbol{drag}}\boldsymbol{=}\boldsymbol{3}\boldsymbol{\pi\mu}\boldsymbol{D}\boldsymbol{u}_{\boldsymbol{0}\boldsymbol{x}}$$

$$\mu=7.3\cdot{10}^{-4}\left[ \frac{N}{m^{2}}\cdot s \right]$$

$$D_{MMP}=2.8\cdot{10}^{-6}\left[ m \right], D_{cell}=10\cdot{10}^{-6}[m]$$

$$u_{0 free particle}=9.8\cdot{10}^{-6}\left[ m/s \right],$$

$$u_{0 cell+1 particle}=5.7\cdot{10}^{-6}\left[ m/s \right],$$

$$u_{0 cell+2 particles}=2.8\cdot{10}^{-6}\left[ m/s \right]$$

Medium viscosity^b^

MMP/cell diameter

MMP/cell velocity

For a free particle

$$\vec{F}_{x drag}=1.8\cdot{10}^{-13}[N]$$

$$\vec{F}_{x magnetic}=\vec{F}_{drag}$$

$$3.9\cdot{10}^{-13}[N]\approx1.8{\cdot10}^{-13}[N]$$

To solve those equations for a cell with one particle and a cell with two particles, substitute the same values except the MMP volume, which should be doubled for the cell with two beads for the magnetic force and the diameter, which should be 10 µm for the viscous drag force. Following the solution, you reach our hypothesis detailed above.

Notes:

1. When two MMPs are conjugated to a cell, the volume should be doubled.
2. According to previuos measurements (Alon et al., 2015).
